# Thermal Barrier Coating on Diamond Particles for the SPS Sintering of the Diamond–ZrO_2_ Composite

**DOI:** 10.3390/ma18040869

**Published:** 2025-02-17

**Authors:** Lucyna Jaworska, Michał Stępień, Małgorzata Witkowska, Tomasz Skrzekut, Piotr Noga, Marcin Podsiadło, Dorota Tyrała, Janusz Konstanty, Karolina Kapica

**Affiliations:** 1 Faculty of Metals Engineering and Industrial Computer Science, AGH University of Krakow, Mickiewicza 30 Av., 30-059 Krakow, Poland; witkowsk@agh.edu.pl (M.W.); dtyrala@agh.edu.pl (D.T.); konstant@agh.edu.pl (J.K.); 2 Faculty of Non-Ferrous Metals, AGH University of Krakow, Mickiewicza 30 Av., 30-059 Krakow, Poland; mstepien@agh.edu.pl (M.S.); skrzekut@agh.edu.pl (T.S.); pionoga@agh.edu.pl (P.N.); karolinazofiakapica@gmail.com (K.K.); 3 Łukasiewicz Research Network, Krakow Institute of Technology, Zakopiańska 73, 30-418 Krakow, Poland; marcin.podsiadlo@kit.lukasiewicz.gov.pl

**Keywords:** barrier coating, diamond, mechanical coating, ZrO_2_, morphology, X-ray diffraction, microstructure

## Abstract

The aim of this work was to obtain a protective ZrO_2_ coating on diamond particles, which was to protect diamond from oxidation and graphitization, enabling sintering of diamond at higher temperatures and lower pressures than its thermodynamic stability in atmospheric conditions. The coatings were obtained by mixing diamond with zirconium and oxidizing in air or oxygen. Mixtures of diamond and 80 wt% zirconium were sintered by SPS method at temperatures of 1250 °C and 1450 °C. To stabilize the tetragonal structure of ZrO_2_, 3 mol% Y_2_O_3_ was added to zirconium before the milling process. The composition of powder phases, morphology, and microstructures of sintered materials were characterized by X-ray diffraction (XRD), scanning electron microscopy (SEM), and energy-dispersive spectrometry (EDS). Diffraction studies show the presence of zirconium monoclinic and tetragonal oxides in coatings, after oxidation in air, and in oxygen. Oxidation in oxygen flow is possible for lower temperatures (75 °C), which results in the presence of unreacted zirconium. In ZrO_2_ doped with yttria after the oxidation process in oxygen, there is no monoclinic ZrO_2._ It is possible to sinter the ZrO_2_–diamond composite at 1250 °C using the spark plasma sintering method without graphitization of the diamond. The sintered material consists of monoclinic and tetragonal ZrO_2_ structures.

## 1. Introduction

Temperature resistance of a diamond is its ability to resist the graphitization and oxidation processes when exposed to an oxidizing atmosphere and at elevated temperatures. For natural diamonds, the direct diamond-to-graphite transition is observed even at 900 °C [1]. At room temperature, diamond is stable at pressures above 1.6 GPa. At temperatures lower than 900 °C, the rate of burning exceeds that of graphitization. At temperatures higher than 900 °C, the graphitization rate increases [2,3]. The graphitization process of synthetic diamond begins at a temperature of approximately 750 °C and intensifies as the temperature increases. There are two main groups of sintered diamond tool materials, these are polycrystalline diamond compacts (PCD) obtained in diamond stable conditions, i.e., in high pressure conditions, with the addition of various binding phases, mainly at temperature above 1500 °C and above 6 GPa [4], but even in these materials graphite is present [5]. Uneven pressure distribution in the pressed material causes tensile stresses on some of the diamond particle surfaces, which, in turn, leads to graphitization of the diamond surface [6]. The second group are materials with cobalt or phases containing iron or nickel matrix, and others, such as diamond saws and grinding wheels, sintered in the graphite stable range (low pressures), up to 950 °C and up to 35 MPa [7]. In this case, graphite is also present on the surface of the diamond extracted from the tool [8]. Thermal resistance of diamond composites depends on the oxidation process more than the graphitization process. CO gas evolution during diamond composite oxidation destroys the integrity of the composite microstructure [9]. As a summary of the diamond graphitization research during heating, R.A. Khmelnitsky et al. suggested the protection of the diamond surface with carbide-forming metals [10]. Diamond companies offer various types of metallic coatings applied to diamond grits to improve their bonding to matrix in a grinding wheel or sawblade segments. This type of coating improves the capacity for diamond retention in the matrix and ensures chemical bonding between the diamond and the matrix. The technology of diamond particles coated by selected carbide forming elements such as Ti, Si, Cr, etc., is an efficient method to enhance the interfacial bonding conditions. Furthermore, it has been proven that carbide coating the diamond surface with a barrier layer is a practical way to protect diamond crystals from degradation at elevated temperatures in oxidizing and corrosive environments [11]. Titanium carbide formed as a result of reaction with carbon from diamond did not stop the graphitization and oxidation processes, but increased the graphitization temperature [12,13,14,15]. Polycrystalline diamond compacts (PCD), with improved thermostability, are synthesized with boron-coated diamond particles, which in the sintering process forms boron carbide B_4_C [16,17].

Various methods of coating diamond with metals and metal carbides have been used, for example a sputtering method, a procedure that involves heating the diamond in a mixture of salt and metals or infiltration by metals with annealing [18,19,20]. Obtaining diamond powders with barrier coatings facilitated the production of carbide-based diamond composite materials. In the work of X.L. Shi et al., the diamond was covered with tungsten using the vacuum vapor deposition method and sintered at a temperature of up to 1280 °C [21]. Other researchers have used diamond powders with SiC coatings for carbide matrix materials [22,23]. From among oxide ceramics, zirconia and alumina are the most important materials. These materials are used as wear-resistant parts, cutting tools, and more. The introduction of diamond may have a positive effect on increasing the thermal shock resistance of these ceramic materials, because diamond is characterized by the highest thermal conductivity of all materials. So far, attempts to sinter the Al_2_O_2_ or ZrO_2_ oxide matrix with the addition of diamond at pressures below 1.6 GPa have failed. Work on sintering these oxides with another superhard material, cubic boron nitride, was successfully completed [24,25]. However, the thermal resistance of boron nitride compared to diamond is twice as high, which allows the use of higher sintering temperatures for a longer time. It was noticed in the work of Z. Pedzich that the stress state caused by the introduction of the second phase into the ZrO_2_ matrix causes a decrease in the share of the monoclinic phase, which has a positive effect on the mechanical properties of the composite [26]. In addition to many other properties expected from thermal barrier coatings, the basic ones are low thermal conductivity and its stability with increasing temperature. These conditions are met by tetragonal zirconium oxide [27]. Tetragonal zirconium oxide is used mainly to protect gas turbines. ZrO_2_ occurs in three allotropic forms: monoclinic, tetragonal, and cubic. At room temperature,, ZrO_2_ has a monoclinic structure, heated up to a temperature of 1170 °C it transforms into the tetragonal structure, and further heating temperature of 2370 °C causes the transformation of the compound into cubic structure [28]. Stabilized tetragonal ZrO_2_ is outstanding in terms of low thermal conductivity and has good thermal shock resistance [29]. The content of the metastable tetragonal phase depends on the share of yttrium oxide and the parameters of the material production process [30]. Currently, the share of Y_2_O_3_ in sintered ZrO_2_ materials is 2 mol% up to 8 mol%. The Y_2_O_3_ content affects the phase composition, the mechanical, thermal, and electrical properties of ZrO_2_, and is optimized with respect to the application of the material [31,32,33]. Yttria-stabilized zirconia (YSZ) exhibit temperature-independent low thermal conductivity at high temperatures [34]. Zirconium oxide in the form of coatings was deposited using the plasma spray technique or by the electron beam–physical vapor deposition process (EB–PVD) [35,36]. In this work, in order to obtain ZrO_2_ coatings constituting a thermal barrier, mechanical mixing of diamond with zirconium in a planetary mill and its subsequent oxidation was used. Materials were sintered using spark plasma sintering. Successful trials of sintering diamond with WC–Co matrices using the SPS method have already been carried out [37,38].

The aim of the work was to obtain a protective ZrO_2_ coating on diamond particles, enabling sintering of diamond with ZrO_2_ matrix at higher temperatures and lower pressures than would result from its thermodynamic stability [39]. The research focused on obtaining a dense composite with the highest possible share of tetragonal ZrO_2_ and diamond.

## 2. Materials and Methods

Commercially available zirconium powders (supplier Kamb Import-Export, Warszawa, Poland) with dedicated grain size below 60 μm and 99.0% purity were used. The chemical composition is presented in Table 1.

**Figure 1:**
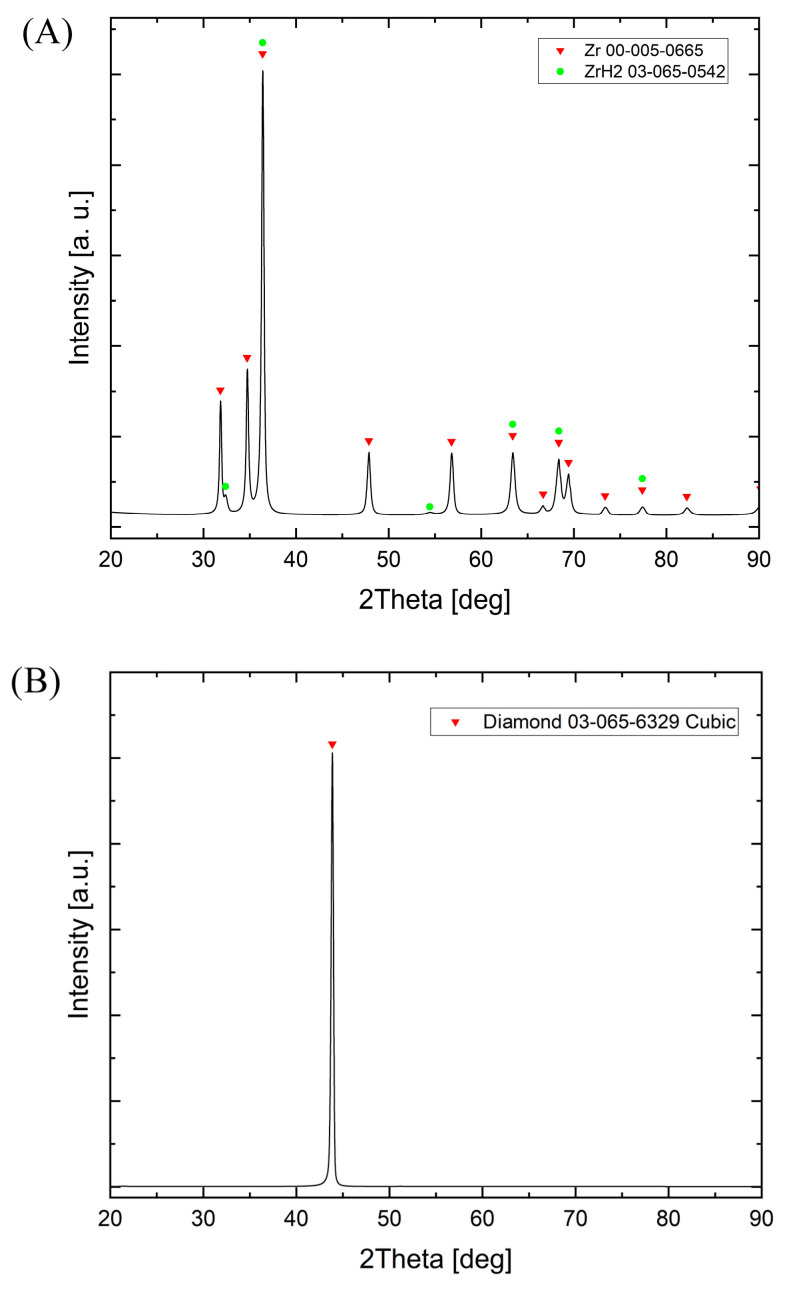
XRD patterns: (**A**) diamond powder (500–350 μm); (**B**) zirconium.

Diamond powder 30/40 and 50/60 (500–350 μm, 300–250 μm, manufactured by Hyperion, Barcelona, Spain) with zirconium was mixed in a Pullverisette 7 planetary mill (FRITSCH GmbH, Idar-Oberstein, Germany). In Figure 1, diffractograms of diamond (A) and zirconium powders (B) are presented.

In order to reduce contamination, a bowl made of zirconium oxide and grinding media in the form of ZrO_2_ balls, 10 mm in size, were used. A total of 1.25 g of zirconium powder was introduced into the diamond powder. The diamond constituted 80 wt% of the powder mixture. Isopropyl alcohol or water were poured into the grinding bowl with powders and grinding balls. The mixing cycle consisted of right–left mixing at a speed of 100 rpm in the following cycles: 15 min of mixing, 5 min of break to cool the system. Four material samples were taken after 30, 60, 90, and 120 h. In this same method with 120 h of mixing, the mixture of 20 wt% of diamond (500–350 μm) and 80 wt% of zirconium was prepared. Another diamond powder (300–250 μm) with 20 wt% (97 mol% Zr + 3 mol% Y_2_O_3_) was mixed at a speed 100 rpm for a total of 30 h. This powder was dried after the mixing. Annealing processes for mixtures were realized in a laboratory furnace (Czylok, Jastrzębie Zdrój, Poland). Heat treatments were carried out using three processes, two in air, one in oxygen. In the first process, diamond powder (500–350 μm) with the zirconium coating was heat treated for 1 h at temperature of 400 °C, in air. In the second process, diamond powder (300–250 μm) with the zirconium coating was kept in a technical oxygen for 30 min (oxygen flow at a level of about 0.5 L/min.), then the heating was started to a temperature of 75 °C. Next, the sample was kept in the furnace for 24 h, after which cooling with the furnace was started. The parameters of zirconium oxidation in air were determined on the basis of studies on the oxidation of materials obtained from zirconium powder [40]. In the third process, diamond (300–250 μm) + 20% (Zr + 3% mol Y_2_O_3_) was heated for 1 h in the furnace to 50 °C, in air flow 0.3 dm^3^/min and next heated in the furnace to 75 °C and kept for 46h, in air flow 0.3 dm^3^/min. After that, material was cooled together with the furnace to ambient temperature. Yttrium oxide Y_2_O_3_ powder (AB134554, GRADE C, particles size D50 0.6–0.9 μm, manufactured by Höganäs AB, Höganäs, Sweden) was used. The mixture of 20 wt% diamond with the zirconia coating and 80 wt% (97 mol% Zr + 3 mol% Y_2_O_3_) was prepared. In order to separate the diamond from the remains of oxidized zirconium, the powders should be separated using a sieve with a mesh smaller than the size of the diamond.

The next stage of the research was the observation of the surface of the obtained mixtures using a scanning electron microscope. Morphologies of powders were carried out on the Hitachi SU-70 scanning microscope with the EDS spectrometer (Hitachi High-Technologies Corporation, Tokyo, Japan). The obtained morphologies of powders made it possible to determine the uniformity of the zirconium coverage of the diamond particles and to determine the most favorable mixing conditions. The coating thickness was detected using a scanning microscope and EDS by breakaway of the layer by long mixing in Al_2_O_3_ powders. The size of the detached ZrO_2_ particles was assessed. Diamond powder with a ZrO_2_ coating was added in an amount of 20% by mass to the Al_2_O_3_ powder (AA-04, Al_2_O_3_, particles size D50 0.5 μm SUMITOMO, Chiyoda City, Japan), then dry-mixed in a TURBULA mixer (Willy A. Bachofen AG, Muttenz, Switzerland) for a period of 6 h. There is a large difference in hardness between ZrO_2_ and Al_2_O_3_, 13 GPa and 18 GPa, respectively, which creates conditions for the breakaway of zirconia from diamond particles by Al_2_O_3_. Of course, ZrO_2_ layer flakes may crumble, but their thickness should not change during the slow, low-energy mixing. SPS apparatus (the FCT Systeme GmbHHP-D5/2, Frankenblick, Germany) was used to the powder sintering. In Table 2, the material compositions and sintering parameters are presented. The diameter of the samples was 20 mm.

Microstructure examination and EDS element distribution of sintered composites were carried out on the Hitachi SU-70 scanning microscope with the EDS spectrometer (Hitachi High-Technologies Corporation, Tokyo, Japan) and the Zeiss Stemi 305 stereoscopic microscope (Zeiss, Jena, Germany). The composite material obtained from 20 wt% diamond (500–350 μm) with the Zr coating after oxidation in air, at 400 °C with 80 wt% ZrO_2_, sintered at 1250 °C was annealed at 800 °C for 30 min, in air. Microstructure tests were carried out using fractures due to the presence of diamond in the samples and the difficulty in the material preparation. Apparent densities were determined by weighing materials in water and in air. X-ray qualitative phase analysis was performed on a Siemens D500 diffractometer (now Bruker, Billerica, MA, USA) using monochromatic radiation from a tube with a copper anode of λKα = 0.154 nm, with steps Δ2θ = 0.04°, counted in the range = 10 s/step.

## 3. Results

### 3.1. Morphology of Diamond Powders

**Figure 2:**
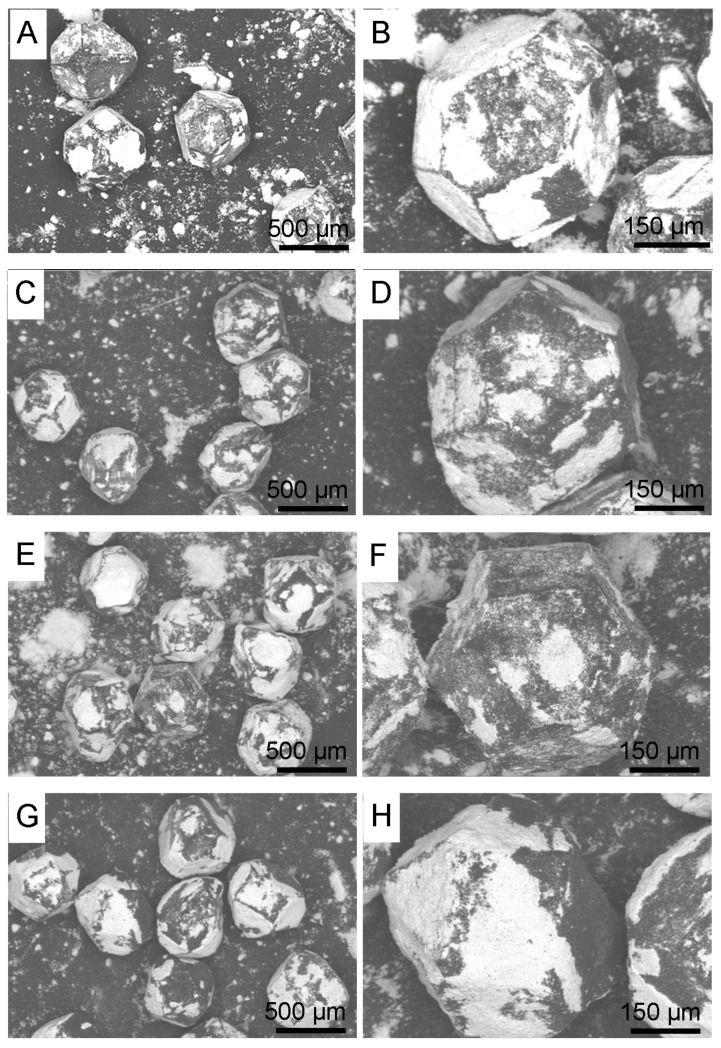
Diamond and zirconium powders mixed with isopropyl alcohol: (**A**,**B**) 30 h; (**C**,**D**) 60 h; (**E**,**F**) 90 h; (**G**,**H**) 120 h.

In Figure 2A–F, morphologies of diamond particles after mixing in a planetary mill in isopropyl alcohol are presented. In Figure 2G,H, morphologies of diamond particles after mixing in a planetary mill in water are shown.

**Figure 3:**
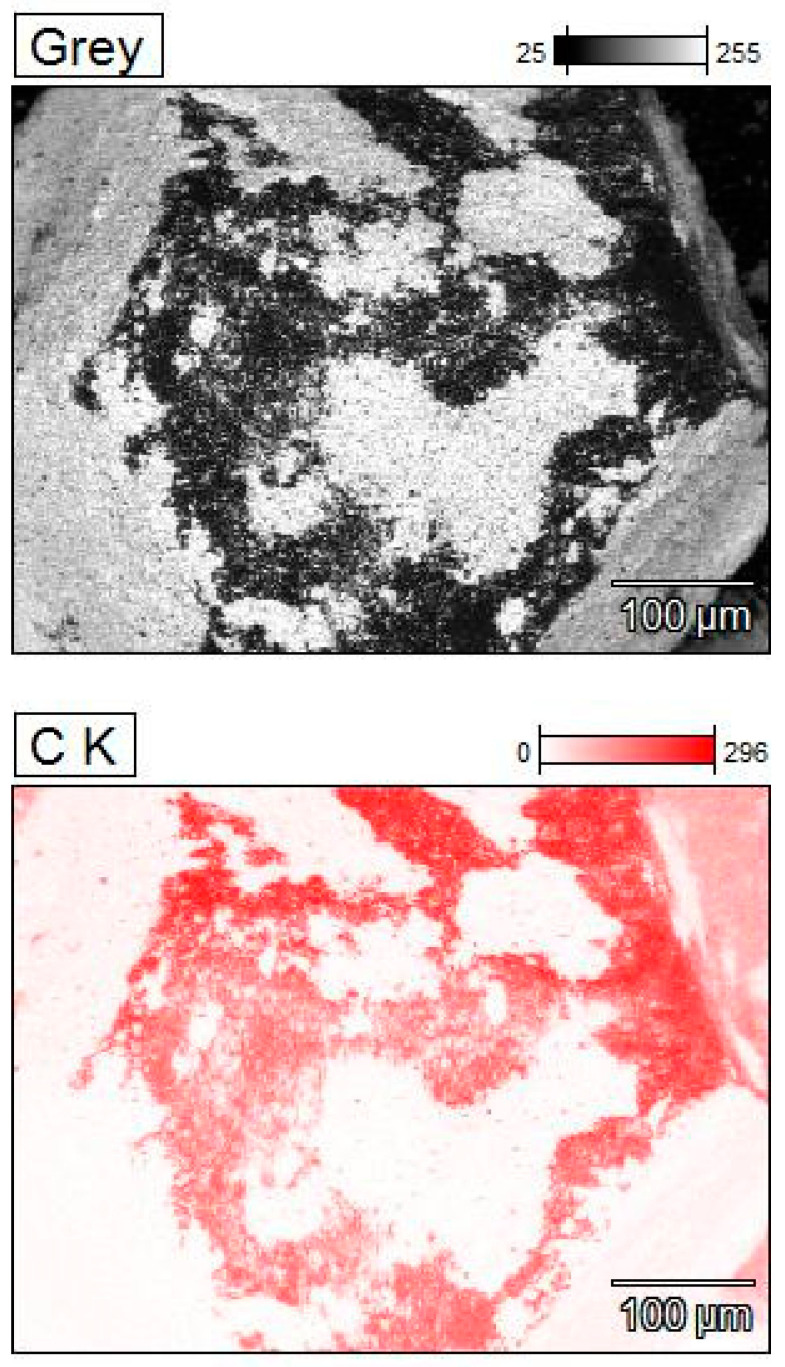

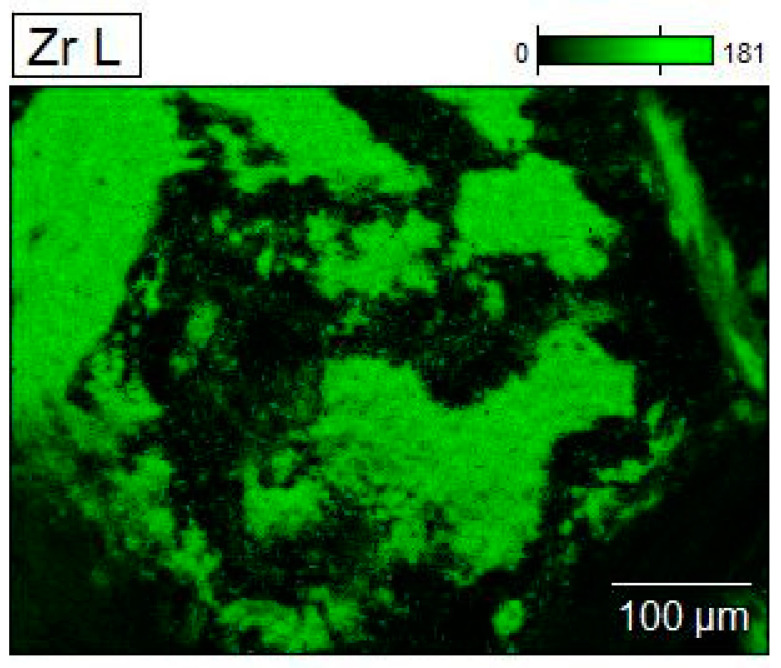
Maps of element distribution for the diamond powder with zirconium after 120 h of the mixing in a isopropyl alcohol.

There is no significant difference between coatings obtained in isopropyl alcohol. The coatings vary in thickness, as indicated by the distribution of zirconium, as shown in Figure 3. A significantly larger amount of zirconium was applied to the surface of the diamond particles during the 120 h mixing in water, as shown in Figure 2G,H.

**Figure 4:**
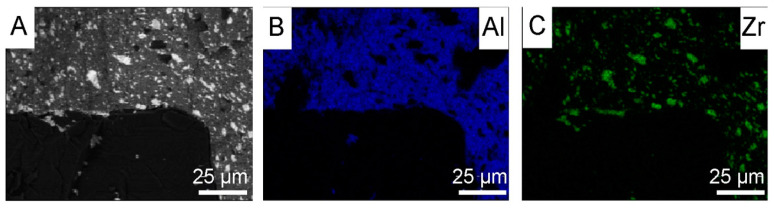
Maps of element distribution for zirconia after 6 h of the mixing in the Al_2_O_3_ powder: (**A**) areas of occurrence of elements with different compositions; (**B**) areas of aluminum; (**C**) areas of zirconium.

Zirconium is plastic, and it covers the diamond surface quite effectively at the beginning of mechanical mixing. During its mixing it oxidizes, and ZrO_2_ particles do not adhere so well to the diamond surface. ZrO_2_ ceramic is stiff and tends to fall off. Therefore, the applied coating does not have the same thickness in every place of the diamond particle. The thickness of the ZrO_2_ coating, obtained in the water mixing was estimated in an indirect way, by measuring the layer peeled off by Al_2_O_3_ powders (Al_2_O_3_ AA-04, D50 0.5 μm, manufactured by SUMITOMO, Japan) during the mixing in alcohol across 6 h. For the sample preparation, the powder was spark plasma sintered at 1450 °C, under pressure 60 MPa, for 5 min. Studies were realized by scanning microscope and EDS. Tests were carried out on the fractured material. The size of the detached ZrO_2_ particles (white particles in Figure 4A and green points in Figure 4C) from diamond range from 1 μm to a maximum of 10 μm; see Figure 4A–C.

### 3.2. Phase Compositions of Diamond Powders

**Figure 5:**
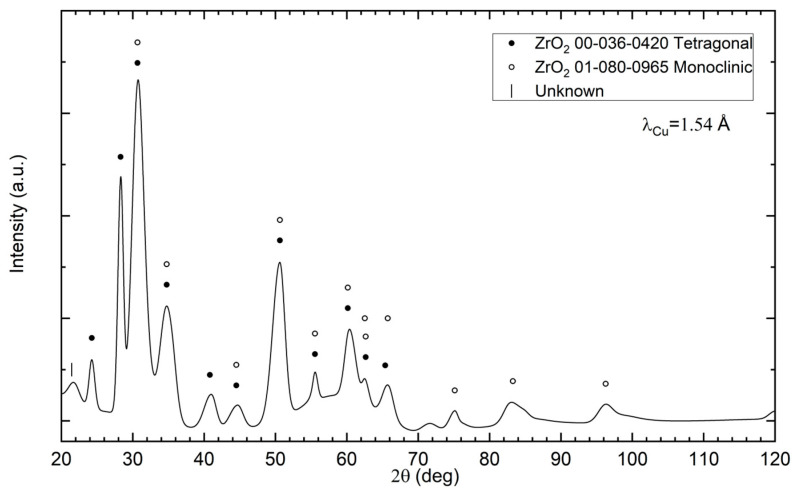
XRD of the obtained diamond (500–350 μm)—zirconium powder after oxidation in air, at 400 °C.

X-ray diffraction study of a mixture of diamond powder and zirconium, presented in Figure 5, shows that the powder sample after oxidation, carried out in air at a temperature of 400 °C, consists of monoclinic and tetragonal varieties of zirconium dioxides. The diffraction pattern contains one uninterpreted peak, which may be due to impurities. The presence of graphite and diamond was not detected, which results in a relatively small surface area of uncoated diamond particles.

**Figure 6:**
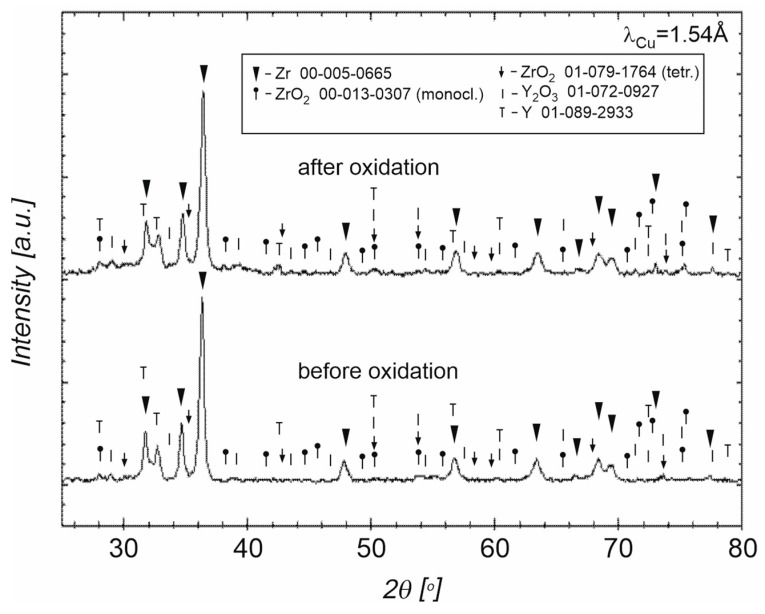
XRD of the obtained diamond (300–250 μm) with zirconium (97 mol% Zr + 3 mol% Y_2_O_3_) coatings before and after the oxidation in oxygen, at 75 °C.

Due to the presence of nitrides in coatings, the oxidation processes of diamond–zirconium mixtures were carried out in oxygen. Nitride–oxide may decompose during further technological processes, e.g., sintering, weakening the adhesion of the barrier coating. For the oxidation in oxygen, it was impossible to use a temperature of 400 °C due to the ignition of the diamond powder with zirconium coatings. The use of higher temperatures than 75 °C caused the mixture to ignite, which is due to the spontaneous combustion of diamond and zirconium. X-ray diffraction studies of diamond powder with ZrO_2_ coating oxidized in oxygen are presented in Figure 6.

In order to stabilize the tetragonal structure of the ZrO_2_ coating, yttrium oxide was added to the mixture. The diffractogram before the oxidizing process for the diamond with the Zr + 3 mol% Y_2_O_3_ coatings is presented. In this same Figure 6, the diffractogram after the oxidizing process at 75 °C for this same diamond with the Zr + 3%mol Y_2_O_3_ is also presented.

Due to the lack of protection of powders from the presence of air during the weighing, pouring, mixing, and drying processes, powder mixtures were already oxidized after the mixing process. The diffractogram of the powder mixture before and after oxidation in oxygen is very similar; see Figure 6. The reason is the activity of zirconium towards oxygen. Phase compositions for both powders are very similar. In the powders before and after the oxidation, there are monoclinic and tetragonal structures of ZrO_2_ but also unreacted zirconium because of the low temperature of zirconium oxidation (75 °C). The mixing in a planetary mill is a high-energy process, therefore, in addition to zirconium and the monoclinic ZrO_2_ structure, the coating also contains a tetragonal phase, which is partially stabilized by yttrium oxide [16]. During the process of annealing the material in oxygen, there is reduction in Y_2_O_3_ to the to the form of yttrium. Zirconium can dissolve significantly more oxygen than yttrium under the oxidation conditions used, up to 35 at%. There is oxygen consumption by zirconium and it could be reason of the yttria reduction [41,42]; see Figure 6.

### 3.3. Powders After SPS

**Figure 7:**
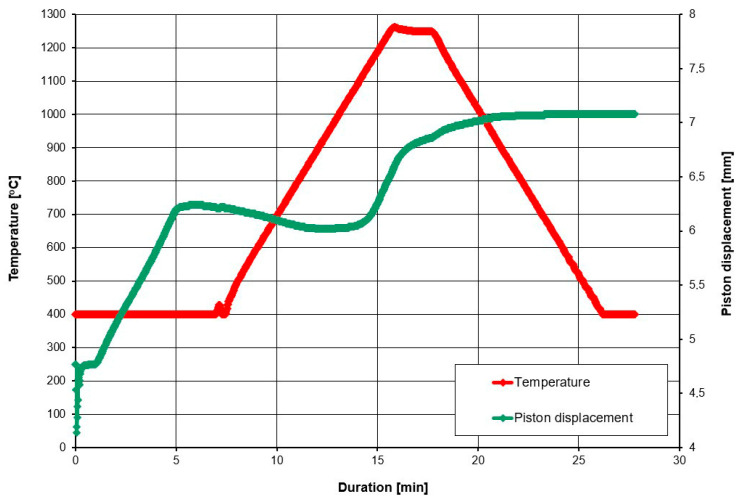
The SPS plungers displacement during sintering for the 20 wt% diamond (500–350 μm) with the 80 wt% ZrO_2_.

The SPS plungers displacement during the sintering of the 20 wt% diamond (500–350 μm) with the 80 wt% ZrO_2_ are presented in Figure 7.

**Figure 8:**
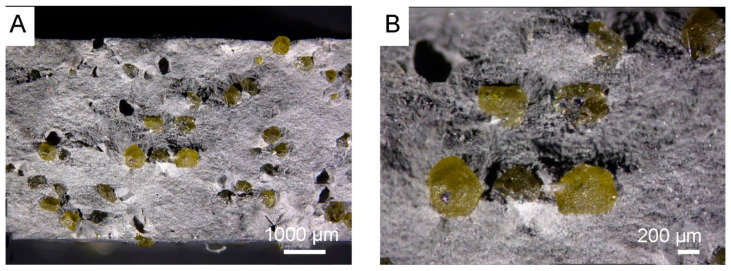
Microstructures of the 20 wt% diamond (500–350 μm) with the Zr coating after oxidation in air, at 400 °C with 80 wt% ZrO_2_, obtained at 1250 °C, using a Zeiss Stemi 305 stereoscopic microscope: (**A**) magnification 10×; (**B**) magnification 50×.

Microstructures of the 20 wt% diamond (500–350 μm) with the 80 wt% ZrO_2_ (mixed in air and annealing at 400 °C), sintered at 1250 °C, are presented in Figure 8A,B, and clearly show the presence of diamond (yellow particles) with rounded edges, resulting from the long milling process. Changes in the volume of the material assessed by the displacement of the pistons show that they occur up to a temperature of about 1000 °C; Figure 7. This indicates that the sintering temperature for this material can be reduced, which is an interesting effect, because ZrO_2_ materials are sintered by the SPS method even at a temperature of 1500 °C [43]. The reason may be the long-term milling of powders in a planetary mill for 120 h and high energy of powders.

**Figure 9:**
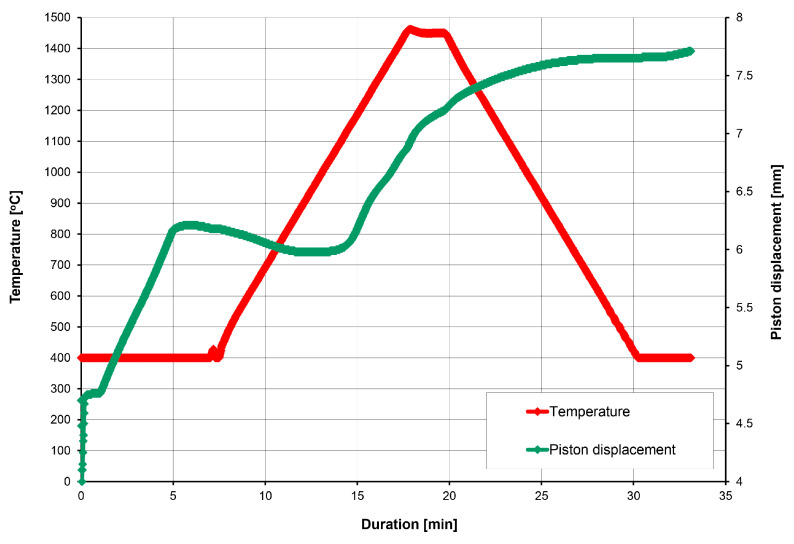
SPS plungers displacement during sintering for 20 wt% of diamond (500–350 μm) with the 80 wt% ZrO_2_.

The SPS plungers displacement during sintering of the 20 wt% diamond (500–350 μm) with the 80 wt% ZrO_2_ are presented in Figure 9.

Changes in volume of the material assessed by the displacement of the pistons (the green curves) show that they occur up to about 1450 °C; see Figure 9. In this case, there is an increase in the volume of the material as a result of diamond transition into graphite, therefore, there is no stabilization of the plungers displacement at 1000 °C, as seen in Figure 7.

**Figure 10:**
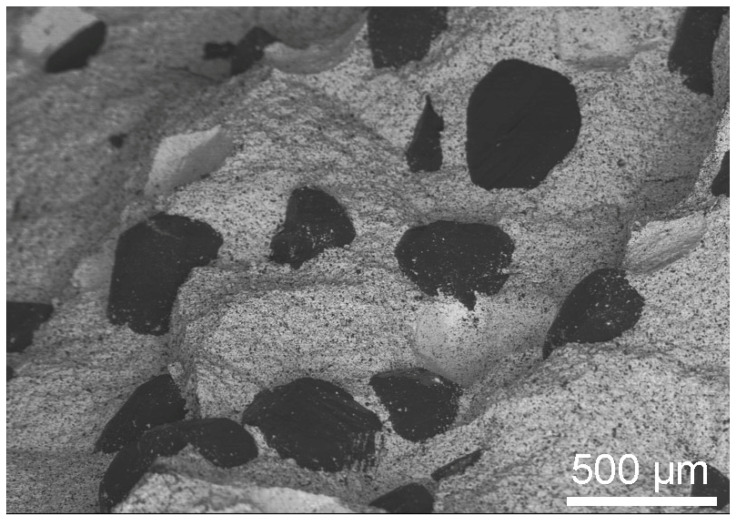
The microstructure of the 20 wt% diamond (500–350 μm) with the 80 wt% ZrO_2_, obtained at 1450 °C.

The SEM microstructure of the 20 wt% diamond (500–350 μm) with the 80 wt% ZrO_2_ (mixed in air and annealing at 400 °C), sintered at 1450 °C is presented in Figure 10.

Apparent densities of materials sintered at 1250 °C and 1450 °C are 4.71 ± 0.0047 g/cm^3^ and 4.25 ± 0.0098 g/cm^3^, respectively. For these materials, it is difficult to determine the theoretical density, taking into account the different phase composition of the materials obtained at different temperatures; therefore, relative densities were not calculated. However, the difference in the density value of the sintered material and that obtained at a higher temperature indicates graphitization of the material, confirmed by the microstructure shown in Figure 10. The black particles are characteristic of a diamond graphitized throughout its entire volume and do not show the planes characteristic of diamond crystals.

**Figure 11:**
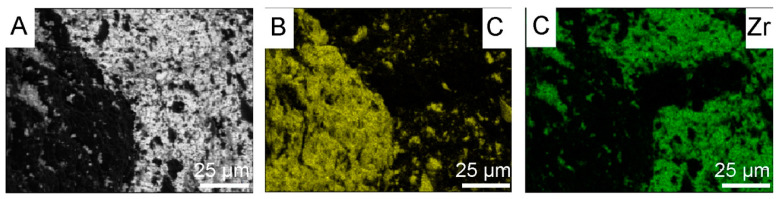
Maps of element distribution for diamond powder with zirconium after 120 h of mixing in the isopropyl alcohol: (**A**) areas of occurrence of elements with different compositions; (**B**) areas of carbon; (**C**) areas of zirconium.

The materials presented in Figure 8 were prepared under conditions of long mixing of Zr powders with diamond for 120 h. As a result, diamond chipping occurred, which can be observed in the microstructures and element maps of carbon and zircon distribution shown in Figure 11B (small yellow particles).

**Figure 12:**
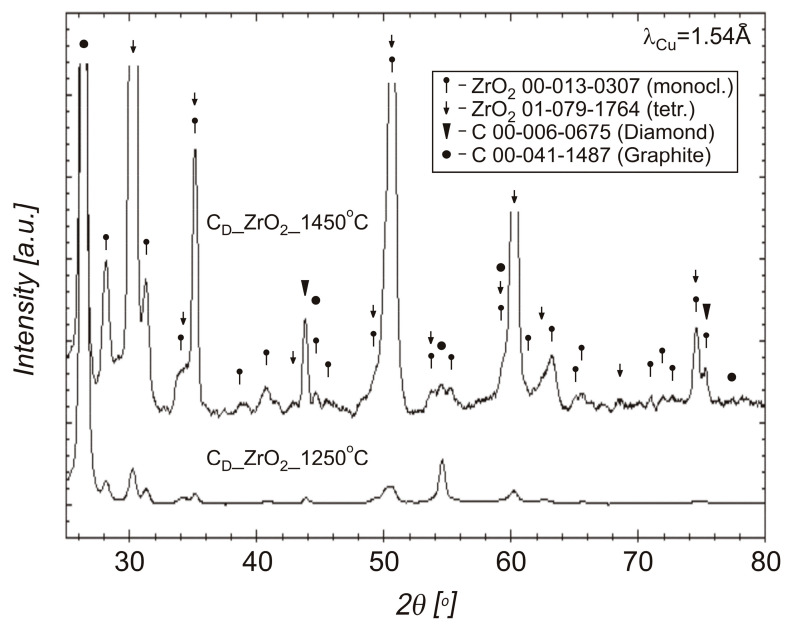
XRD patterns of the SPS sintered at 1250 °C and 1450 °C, materials from the diamond (500–350 μm, 20 wt%) with zirconium powders (80 wt%) after oxidation in air, at 400 °C.

X-ray diffraction studies of sintered materials from 20 wt% diamond (500–350 μm) with 80 wt% ZrO_2_, obtained at 1250 °C and 1450 °C, using the SPS method are presented in Figure 12.

**Figure 13:**
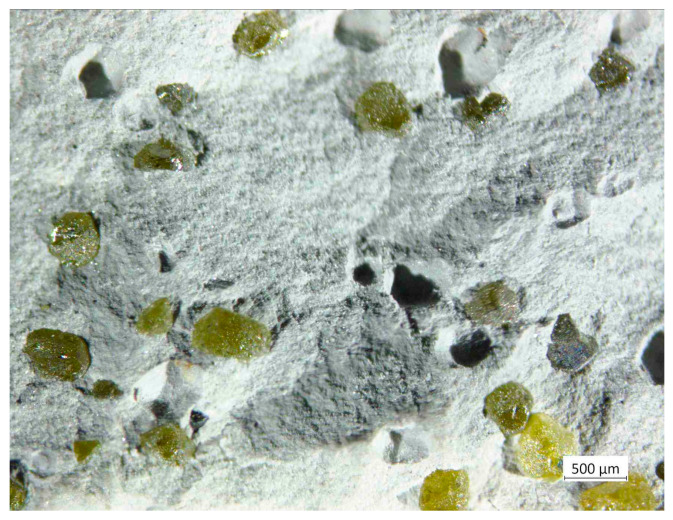
Microstructures of the 20 wt% diamond (500–350 μm) with the composite, obtained at 1250 °C, after the annealing at 800 °C for 30 min, using a Zeiss Stemi 305 stereoscopic microscope.

Microstructure of this material after annealing at 800 °C in air, for 30 min. is presented in Figure 13.

**Figure 14:**
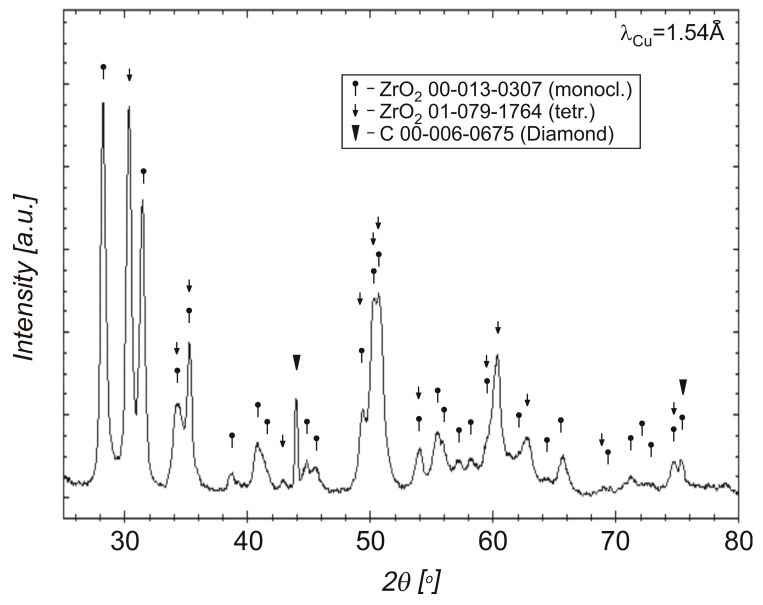
XRD of the SPS sintered material from the diamond (500–350 μm, 20 wt%) with zirconium powders (80 wt%) after the annealing at 800 °C in air.

After heating up to 800 °C, particles of non-graphitized diamond are visible in the composite, which confirms the thermal resistance of this material; see Figure 14. The temperature of the annealing at 800 °C corresponds to the working temperature of the tools without cooling. The phase composition of the diamond composite material after the annealing is presented in Figure 14.

The XRDs presented in Figure 14 shows that after heating the material at 800 °C (the composite sintered at 1250 °C), there are diamond and tetragonal ZrO_2_ in the material.

**Figure 15:**
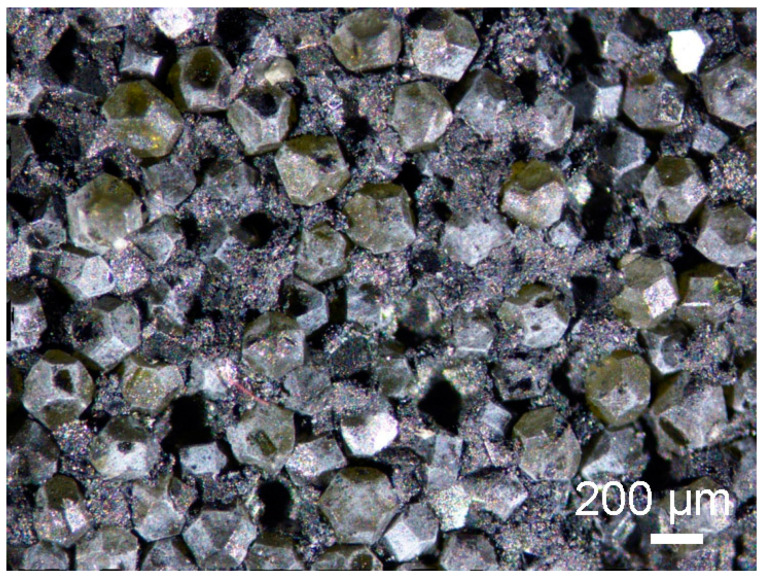
Diamond (300–250 μm) with 20 wt% ZrO_2_ + 3% mol Y_2_O_3_ coatings (heated for 1 h in the furnace to 50 °C and next heated in the furnace to 75 °C, in air) after annealing using SPS at 1250 °C, 60 MPa, 2 min, using a Zeiss Stemi 305 stereoscopic microscope.

Figure 15 shows diamond powders with ZrO_2_ coating after annealing at 1250 °C, using the SPS apparatus. The light color and the shape of the powders inside indicates the presence of diamond.

## 4. Discussion

A shortened time of mixing zirconia with diamond to 30 min was used. In the process of oxidation of zirconium on the surface of diamond particles, two phenomena are important. The first is the oxidation of zirconium. The second is the oxidation and graphitization of diamond. As a result of the thermal treatment of powder in air, at 400 °C, diffraction study shows the presence of monoclinic and tetragonal zirconium oxide. Stabilized tetragonal ZrO_2_ is outstanding in terms of low thermal conductivity and has good thermal shock resistance and is favorable for use as a thermal barrier [29]. The powders were sintered at temperatures of 1250 °C and 1450 °C. In both cases, the basic phase after the SPS sintering process was mainly the tetragonal ZrO_2_ phase. At ambient pressure and temperature above 1170 °C, the monoclinic ZrO_2_ phase transforms to a tetragonal phase, while at ambient temperature and under high pressures, several orthorhombic phases are also observed. The first orthorhombic phase appears between 3.0–11.0 GPa [44].

In the SPS processes presented in Table 2, 60 MPa was used. The use of pressure is associated with an increase in mechanical stresses in materials. It was noticed in the work of Z. Pedzich that the stress state caused by the introduction of the second phase into the ZrO_2_ matrix causes a decrease in the share of the monoclinic phase, which has a positive effect on the mechanical properties of the composite [26]. According to Chevalier et al., tetragonal prime zirconia can be formed by heating to high temperature where the cubic phase is stable and cooling, or by rapid deposition from the vapor or liquid [45]. The SPS method is a non-equilibrium sintering method. The process is carried out at a relatively high heating and cooling rate, and, additionally, it is carried out under pressure; these conditions affect the presence of metastable phases under normal conditions [46]. SPS conditions probably influence the presence of the tetragonal phase in the material. In both cases, for powders oxidized at 400 °C and sintered at 1250 °C and 1450 °C, the presence of graphite and diamond was demonstrated in the materials. Graphite in the form of a thin layer is located on the surface of the crystals. However, the apparent density of the composites indicates that most of the graphite content was in the sample sintered at 1450 °C; see Figure 10. The presence of diamond was confirmed by the organoleptic method, confirming the presence of diamond based on the characteristic light reflection; see Figure 8 and Figure 13. The presence of thin layers of graphite on the surface of a diamond is a common phenomenon accompanying the densification processes even under high pressure conditions. In the case of the material sintered at 1450 °C, the graphite content is higher, as indicated by the black color of the particles in the sample and the lower value of the apparent density, as seen in Figure 10. Diffraction studies of composite materials in which the reinforcing phase is in the form of diamond particles, spaced apart from each other, coated by ZrO_2_ are very difficult. Diamond particles are well bonded to zirconium oxide; see Figure 8.

Mixing diamond with zirconium in the presence of ZrO_2_ grinding balls for 120 h causes chipping of the diamond edge and the appearance of fine diamond particles in the ZrO_2_ matrix, as shown in Figure 11B. Due to the similar degree of coverage of diamond particles by zirconium during the mixing process, which is visible in the Figure 2G,F, the mixing time was shortened to 30 h. Figure 15 shows that the edges of the diamond for the 30 min mixing are not chipped.

Due to the presence of monoclinic ZrO_2_ in the phase composition of the powder after mixing in the planetary mill and after the oxidation in air at 400 °C, which is visible in Figure 5, yttrium oxide (3 mol%) was added into the Zr to stabilize the tetragonal form of ZrO_2_. In the case of using powders with a coating containing a large share of the monoclinic ZrO_2_ for sintering, changes in the volume of the ZrO_2_ coating can be expected during powder sintering and material cooling, due to phase transformation and changes in the volume of the ZrO_2_ unit cell, which can result in the coating falling off. Powders with yttria after the mixing and after oxidation in oxide are composed mainly of tetragonal and monoclinic structures of ZrO_2_; see Figure 6. The phase composition of the material sintered without the use of yttrium oxide stabilizer does not change after heating at a temperature of 800 °C (corresponding to the operation of cutting tools without cooling), which is confirmed by the diffraction pattern shown in Figure 14. Diffractograms for mixed powders and after oxidation with the additive of Y_2_O_3_, presented in Figure 6 are very similar. The difference is the presence of reduced yttrium in the powders after oxidation at 75 °C. Oxidation in oxygen flow is possible for lower temperatures (75 °C), due to the possibility of spontaneous combustion of the zirconium and diamond mixture, which results in the presence of unreacted zirconium. For this reason, it is more advantageous to oxidize zirconium in air at a temperature of 400 °C. Studies indicate the possibility of using oxide coatings as barrier coatings on diamond, enabling sintering of materials containing diamond, up to a temperature of 1250 °C. In the case of using pressure methods during the SPS, a tetragonal ZrO_2_ structure appears, which is advantageous in terms of thermal conductivity, without the use of a stabilizer in the form of yttria oxide. Zirconium coatings of more uniform thickness can be obtained by other methods (for example the PVD method) and these methods can be used in future work to obtain diamond powders with zirconium oxide barrier coatings. In this case, it is worth considering the presence of a tetragonal ZrO_2_ stabilizer in the form of, for example, yttria oxide. The diamond–ZrO_2_ composite is a material that is difficult to use in machines, as evidenced by the difficulties in preparing specimens for testing in this work. In the future, it may be a tool material intended for cutting and drilling stone.

## 5. Conclusions

The obtained ZrO_2_ barrier coatings allow us to obtain a composite material with a ZrO_2_ matrix reinforced with diamond particles, sintered by the SPS method, at a temperature of 1250 °C. Material after SPS has a favorable matrix composition that is composed of tetragonal and monoclinic structures of ZrO_2_.

The mechanical mixing of diamond powders with zirconium powders and the oxidation process do not ensure obtaining a coating of uniform thickness.

Diamond with zirconium and 3 mol% of Y_2_O_3_ powders after the high-energy milling in the planetary miller, before oxidation, partially contain the advantageous tetragonal structure of zirconium oxide.

## Figures and Tables

**Table 1:** Chemical composition of zirconium powder used for testing.

Zirconium (Zr) Powder—CA-% Metal Basic	
Cl	<0.02
Fe	<0.2
Ca	<0.02
Sn	<0.3
Hf	<0.5
Al	<0.05
Mg	<0.1
Si	<0.08
H—75 µm and 45 µm size	<0.1
H—2 µm size	=0.5–1 wt%—protected
Zr	99%

**Table 2:** Composition of diamond–ZrO_2_ materials and parameters of their sintering using the SPS method, in argon.

Materials	Composition [wt%]	Mixing Duration [h]	Temperature of Sintering [°C]	Pressure of Sintering [MPa]	Duration of Sintering [min]
Diamond 500–350 μm + Zr	20 80	120	1250	60	2
Diamond 500–350 μm + Zr	20 80	120	1450	60	2
Diamond 300–250 μm + Zr	80 20	30	1250	60	2

## Data Availability

The original contributions presented in this study are included in the article. Further inquiries can be directed to the corresponding author.
